# Suppression of Interface Traps and Improved Breakdown in Recessed-Gate AlGaN/GaN MISHEMTs Using Low-Temperature Nitrogen Passivation

**DOI:** 10.1021/acsomega.5c10644

**Published:** 2026-04-21

**Authors:** Hsin-Chu Chen, An-Chen Liu, Cheng-Hsien Lin, Sheng-Yao Chou, Ting-Chang Chang, Tsung-Sheng Kao, Hao-Chung Kuo

**Affiliations:** † Institute of Advanced Semiconductor Packaging and Testing, 34874 National Sun Yat-sen University, Kaohsiung 804201, Taiwan; ‡ Institute of Innovative Semiconductor Manufacturing, National Sun Yat-sen University, Kaohsiung 804201, Taiwan; § Department of Photonics, College of Electrical and Computer Engineering, 34914 National Yang Ming Chiao Tung University, Hsinchu 30010, Taiwan; ∥ Department of Physics, National Sun Yat-sen University, Kaohsiung 804201, Taiwan; ⊥ Department of Materials and Optoelectronic Science, National Sun Yat-sen University, Kaohsiung 804201, Taiwan; # Department of Electronic Engineering, Chung Yuan Christian University, Taoyuan 32023, Taiwan; ¶ Semiconductor Research Center, Hon Hai Research Institute, Taipei 114699, Taiwan

## Abstract

Recessed-gate AlGaN/GaN MISHEMTs are promising candidates for power electronics but often suffer from interface defects introduced during gate recessing, which degrade device performance and reliability. In this work, a supercritical fluid nitrogen passivation (SCFNP) technique is developed to address this limitation. The process is carried out at 180 °C under high pressure and enables efficient defect repair at both the Al_2_O_3_/AlGaN interface and the recessed-gate surface. Devices treated with SCFNP exhibited significantly improved electrical performance, including a positive threshold voltage (*V*_TH_) shift to +2.2 V (extracted at *I*_D_ = 1 mA/mm), an increased maximum drain current of 600 mA/mm, a substantially reduced gate leakage current of 9.9 × 10^–8^ mA/mm, and a high on/off current ratio of 5.0 × 10^9^. The gate leakage current decreased by nearly 2 orders of magnitude, while the OFF-state breakdown voltage (BV) increased by 27.8% (from 557 to 712 V). Dynamic on-resistance (*R*_ON_) measurements further revealed a 25.6% reduction in current collapse under high-voltage stress. TCAD simulations corroborate these experimental results by demonstrating that reduced interface trap density suppresses peak electric fields at the drain edge, thereby validating the observed BV enhancement mechanism. Overall, SCFNP represents a low-temperature and scalable postfabrication passivation strategy for reliable GaN power devices.

## Graphical abstract

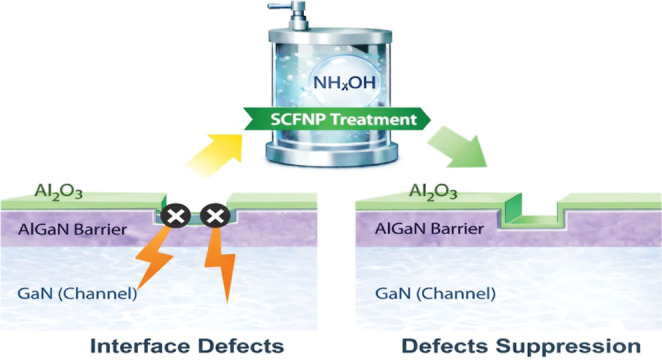

## 1 Introduction

Gallium nitride high-electron-mobility transistors (GaN HEMTs) have attracted considerable attention for high-power and high-frequency applications owing to their wide bandgap, high breakdown field, high electron mobility, low on-resistance, and fast switching capability.^1^ Among the various configurations, normally off GaN HEMTs are particularly desirable because they reduce switching losses, improve operational safety, and simplify gate-driver circuit design, thereby enabling their integration into in power conversion systems.^2^ Commonly adopted approaches to realize normally off operation include cascode configurations,^3^ p-GaN gate HEMTs,^4,5^ and recessed gate GaN MISHEMTs.^6,7^ In cascode designs, a Si MOSFET is combined with a normally on GaN HEMT to achieve a normally off characteristic. However, this hybrid configuration inevitably introduces parasitic inductance and capacitance, which can slow switching transients and induce voltage overshoots that cause false turn-on. In p-GaN gate HEMTs, Mg doping is employed to deplete the two-dimensional electron gas (2DEG) beneath the gate, thereby shifting the *V*_TH_ positively and providing a robust solution. Nevertheless, drawbacks such as increased gate capacitance and possible Mg diffusion toward the GaN interface raise concerns regarding long-term reliability.^8,9^ Recessed-gate MISHEMTs offer advantages including reduced gate leakage and enhanced *V*_TH_ controllability. However, their fabrication typically involves plasma-assisted deposition and etching processes, during which high-density plasma exposure can induce surface dangling bonds, nitrogen vacancies, and other plasma-related defects on the AlGaN surface. These defects significantly increase the density of trapping states, leading to current collapse, dynamic on-resistance (*R*_ON_) degradation, and compromised device reliability.^10,11^ To mitigate etching-induced surface damage, thermal annealing has been widely employed to reduce gate degradation and eliminate carbon- or oxygen-related defects. Conventional postetch annealing is typically performed at elevated temperatures ranging from 400 to 800 °C, followed by controlled cooling. However, such high-temperature treatments may induce impurity diffusion, dielectric crystallization, and thermally generated stress-related defects, which are detrimental to interface stability and long-term device reliability.^12,13^ In addition to thermal annealing, plasma-based surface treatments, such as N_2_ or NH_3_ plasma nitridation, have also been extensively investigated to repair recessed-gate damage and suppress interface states. Although these plasma-assisted approaches can partially passivate surface defects, they inevitably introduce additional plasma exposure, which may cause secondary damage, nonuniform nitrogen incorporation, and passivation effects limited primarily to near-surface regions.^14^ More importantly, for recessed-gate MISHEMTs, the damaged regions are spatially confined beneath the gate dielectric and along the recessed sidewalls, where conventional plasma and surface-limited treatments inherently lack sufficient penetration capability to uniformly passivate buried defect sites. Wet chemical passivation methods, while plasma-free, generally suffer from limited penetration depth and unstable passivation behavior under high electric field stress.^15^

In this work, to specifically address the limitations of conventional passivation techniques in recessed-gate MISHEMTs, a low-temperature nitrogen passivation technique based on supercritical fluid (SCF) technology is proposed. Supercritical fluids (SCFs) exhibit liquid-like density and gas-like viscosity, providing superior permeability and solubility that enable dissolution and transport of reactive nitrogen species into solid materials at relatively low temperatures.^16–19^ The supercritical fluid nitrogen passivation (SCFNP) process effectively passivates surface dangling bonds on AlGaN and penetrates into the AlGaN/GaN heterostructure following recessed-gate etching, without introducing additional plasma-induced damage. As a result, SCFNP enhances both the electrical performance and reliability of recessed-gate MISHEMTs, as confirmed by DC and *C*–*V* characterization, OFF-state breakdown voltage measurements, and dynamic *R*_ON_ stability tests. Furthermore, TCAD simulations incorporating different interface defect densities reveal that SCFNP suppresses trap-assisted field modulation, leading to a pronounced reduction in the peak electric field at the drain-side gate edge. This simulation result is consistent with experimental observations, where devices treated with SCFNP exhibit a 27.8% enhancement in OFF-state breakdown voltage. These findings demonstrate, for the first time, a direct correlation between SCFNP-induced interface defect suppression, electric-field redistribution, and BV improvement in recessed-gate MISHEMTs. Collectively, this study establishes SCFNP as a fundamentally distinct, low-temperature, nonplasma passivation paradigm with deep-penetration capability for mitigating recessed-gate damage in GaN power devices.

## 2 Experimental Section

The normally off AlGaN/GaN MISHEMT with a recessed-gate structure was fabricated on a 6-in. commercial Si substrate using metal–organic chemical vapor deposition (MOCVD). The epitaxial stack consisted of a 1 nm GaN cap layer, a 25 nm Al_0.25_Ga_0.75_N barrier layer, a 1 nm AlN interlayer, a 300 nm undoped GaN channel layer, and a 4 μm GaN buffer layer, as illustrated in Figure 1(a). Hall measurements performed on the as-grown epitaxial wafer revealed a two-dimensional electron gas (2DEG) sheet carrier density of 9.8 × 10^12^ cm^–2^ and an electron mobility of 1650 cm^2^/V·s at room temperature. The device fabrication process began with mesa isolation defined by inductively coupled plasma reactive ion etching (ICP-RIE). Ohmic contacts for the source and drain electrodes were subsequently formed using Ti/Al/Ni/Au metal stacks (25/125/45/75 nm), followed by rapid thermal annealing at 825 °C for 30 s in a nitrogen (N_2_) ambient. The resulting channel sheet resistance (*R*_sh_) was 385 Ω/sq, and the contact resistance (*R*_c_) was evaluated to be 0.6 Ω·mm using the transfer length method (TLM). The recessed-gate structure was realized using plasma-enhanced atomic layer etching (PEALE), which employs sequential oxidation and low-power Cl_2_/BCl_3_ plasma removal steps to achieve nanoscale depth control with minimized plasma-induced damage. Compared with conventional continuous dry etching, this quasi-self-limiting ALE process effectively suppresses ion bombardment and ultraviolet-induced surface degradation, resulting in an ultrasmooth recessed surface and improved interface quality, as previously verified by AFM measurements (*R*_a_ of 0.4 nm).^20^ After the recessed-gate structure was formed, SCFNP was carried out in a high-pressure reaction chamber at 180 °C and 3000 psi for 1 h, using a mixed precursor of 25% aqueous ammonia and CO_2_ gas. Carbon dioxide, commonly employed in SCF processes, is nontoxic, chemically stable, and decomposes only at extremely high temperatures (3200–4600 K),^21^ thereby ensuring that no carbon contamination occurs under the present processing conditions (180 °C). After the SCFNP treatment, the chamber was depressurized, and the device was removed. A post-SCFNP annealing step was subsequently performed to eliminate residual water molecules and thereby prevent oxidation-induced performance degradation. Following this step, a 15 nm Al_2_O_3_ dielectric layer was deposited by atomic layer deposition (ALD) at 350 °C. The fabrication process was completed with back-end-of-line (BEOL) steps, including gate field-plate integration, SiN_*x*_ passivation, and contact opening formation. Subsequently, a Ni/Au metal stack (50/300 nm) was deposited to form the pad electrodes. The recessed-gate MISHEMT had device dimensions of *L*_g_ = 3 μm, *L*_gs_ = 5 μm, *L*_gd_ = 10 μm, and *W*_g_ = 100 μm, as illustrated in Figure 1a,b. These dimensions were intentionally designed for high-voltage lateral GaN power switching applications, with emphasis on breakdown voltage and device reliability rather than aggressive gate-length scaling for RF performance.

**Figure 1:**
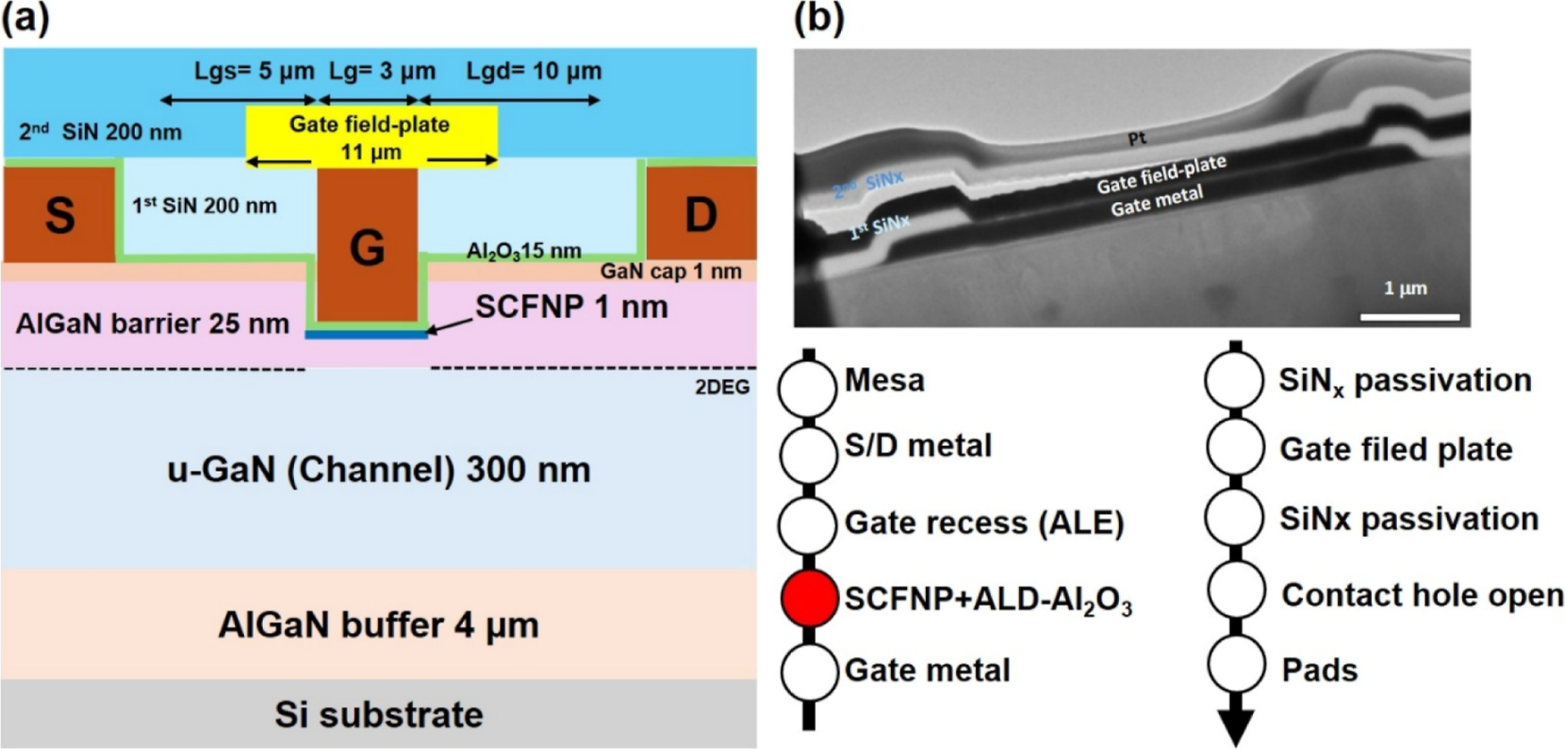
(a) Cross-sectional schematic of the recessed-gate AlGaN/GaN MISHEMT after SCF nitrogen passivation. (b) TEM cross-section and fabrication process flows.

To investigate the chemical and structural modifications induced by SCFNP, complementary X-ray photoelectron spectroscopy (XPS) and cross-sectional transmission electron microscopy (TEM) analyses were performed, as summarized in Figure 2. Figure 2a presents the Ga 2p core-level spectra for samples with and without SCFNP treatment. The deconvoluted spectra reveal a reduction in Ga–O bonding from 37% to 30% and a corresponding increase in Ga–N bonding from 63% to 70% after treatment. These results indicate that SCFNP effectively suppresses native surface oxidation while promoting the preservation or reformation of Ga–N bonds, which are critical for maintaining the chemical integrity of the AlGaN surface. The atomic percentage of N 1s increases from 47.9 at. % to 59.3 at. % after SCFNP treatment, while the O 1s concentration decreases from 23.5 at. % to 10.3 at. %, indicating a reduction of oxygen-related surface species and enrichment of nitrogen-related bonding. A schematic illustration of the proposed surface reaction mechanism is shown in Figure 2b. The schematic in Figure 2(b) illustrates a phenomenological reaction model consistent with established SCF-assisted nitrogen passivation frameworks^22^ rather than a directly measured reaction kinetic pathway. During the SCFNP process, ammonium ions (NH_2_^+^) are generated and interact with surface dangling bonds, weakening Ga–O bonds and facilitating the reformation of Ga–N bonds through nitrogen functionalization. This reaction pathway is proposed to improve surface stoichiometry and reduce interface trap densities at the AlGaN surface. To further examine the structural modifications, cross-sectional TEM images of the recessed-gate region before and after SCFNP treatment are shown in Figure 2c–e. As illustrated in Figure 2c, the remaining AlGaN barrier thickness after gate recess etching is approximately 3.14 nm. A comparison between Figure 2d and e reveals the formation of an ultrathin interfacial layer (0.91 nm) at the Al_2_O_3_/GaN interface following SCFNP treatment. This layer is absent in the untreated device (Figure 2d) but is clearly observed after processing (Figure 2e), indicating that SCFNP induces an interfacial modification at the dielectric/semiconductor interface. Although XPS provides direct chemical evidence of bonding changes, the ultrathin nature of this interfacial layer limits the spatial resolution of energy-dispersive X-ray spectroscopy (EDS), making direct compositional identification challenging. Nevertheless, the presence and morphology of the interfacial layer are clearly confirmed by TEM. When considered together with the electrical and spectroscopic results discussed above, these observations are consistent with an improved interface condition following SCFNP treatment.

**Figure 2:**
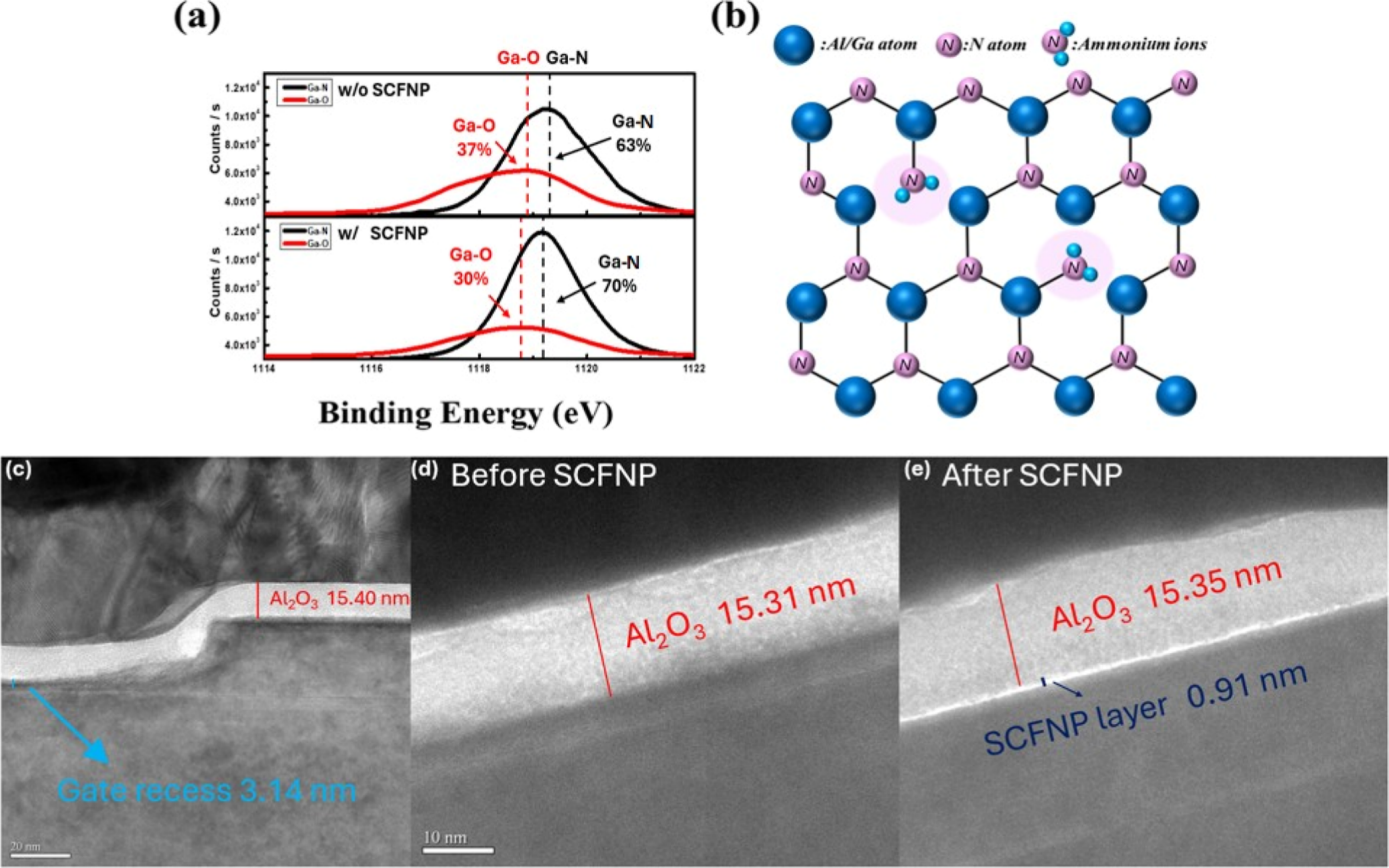
(a) XPS Ga 2p spectra of devices with and without SCFNP, showing reduced Ga–O and increased Ga–N bonding. (b) Proposed surface reaction mechanism illustrating NH_2_^+^ interaction with dangling bonds to restore Ga–N bonds. (c) Cross-sectional TEM image of the recessed-gate region showing remaining AlGaN thickness (3.14 nm). (d) TEM image of an untreated device without an interfacial layer. (e) TEM image of the SCFNP-treated device showing the formation of an ultrathin (0.91 nm) interfacial layer at the Al_2_O_3_/GaN interface.

## 3 Result and Discussion

After device fabrication, the transfer characteristics of recessed-gate AlGaN/GaN MISHEMTs with and without SCFNP were analyzed at room temperature. Figure 3a shows the normalized transfer curves measured at *V*_DS_ = 0.1 V in the linear scale. The SCFNP-treated device exhibited a higher drain current at *V*_G_ = 10 V compared to the untreated counterpart. Specifically, device characterization revealed a 43% increase in the maximum drain current (*I*_D,max_) and a 2.3% improvement in the peak transconductance (*G*_m,max_) after SCFNP. As shown in the semilogarithmic transfer characteristics in Figure 3(b), a pronounced positive *V*_TH_ shift is observed after SCFNP treatment. The SCFNP-treated device exhibits a *V*_TH_ of +2.2 V, whereas the untreated device shows a negative *V*_TH_ of −1.8 V, both extracted at a drain current criterion of 1 mA/mm. This corresponds to a net positive shift of Δ*V*_TH_ of 4.0 V. The substantial *V*_TH_ shift observed after SCFNP treatment reflects an additional modulation of gate electrostatics under an identical recessed-gate geometry, which is consistent with modified interface-related charge conditions introduced by SCFNP. The transfer characteristics measured at *V*_DS_ = 10 V and plotted on a semilogarithmic scale are presented in Figure 3b and summarized in Table 1. The reduction of AlGaN barrier thickness induced by controlled gate recess modifies the polarization-induced charge and reduces the 2DEG density under the gate, leading to a positive shift in *V*_TH_. While recess etching may introduce additional interface scattering, the preserved drain current characteristics after SCFNP treatment indicate effective suppression of recess-induced interface traps, consistent with previously reported thickness-dependent 2DEG modulation behavior.^20^ The SCFNP-treated device exhibited a higher *I*_D,max_ of 600 mA/mm compared to 524 mA/mm for the untreated device, together with a substantially reduced gate leakage current of 9.9 × 10^–8^ mA/mm, which is more than 2 orders of magnitude lower than that of the untreated device (4.1 × 10^–6^ mA/mm). Notably, both the gate leakage current and drain leakage current were significantly reduced after SCFNP, highlighting the effectiveness of nitrogen-based supercritical passivation in suppressing surface- and interface-related leakage pathways that are sensitive to interfacial defect states. As a result, the on/off current ratio improved by nearly 2 orders of magnitude, reaching 5.0 × 10^9^ for the SCFNP-treated device in contrast to 8.6 × 10^7^ for the untreated device. In addition, the subthreshold swing (SS) was reduced from 152.8 mV/dec to 132.5 mV/dec, indicating reduced interface trap-related effects at the AlGaN/GaN interface. The reduction in SS further confirms that SCFNP effectively passivates interface states, thereby improving gate controllability and reducing the influence of interface traps. This substantial suppression of leakage current and improvement in switching characteristics are critical for achieving high electrical performance in recessed-gate AlGaN/GaN MISHEMTs. While advanced fabrication techniques such as low-damage ALE have been proposed to minimize plasma-induced damage during gate recessing, they require stringent process control and may not fully eliminate surface trap formation. In contrast, SCFNP provides a postetch surface defect passivation pathway that suppresses residual traps and restores interface quality. Importantly, these enhancements are obtained without adversely affecting essential device parameters such as *V*_TH_ or *G*_m_, thereby highlighting the significance of SCFNP as a complementary surface and interface engineering strategy for achieving high-performance and reliable GaN-based devices.

**Figure 3:**
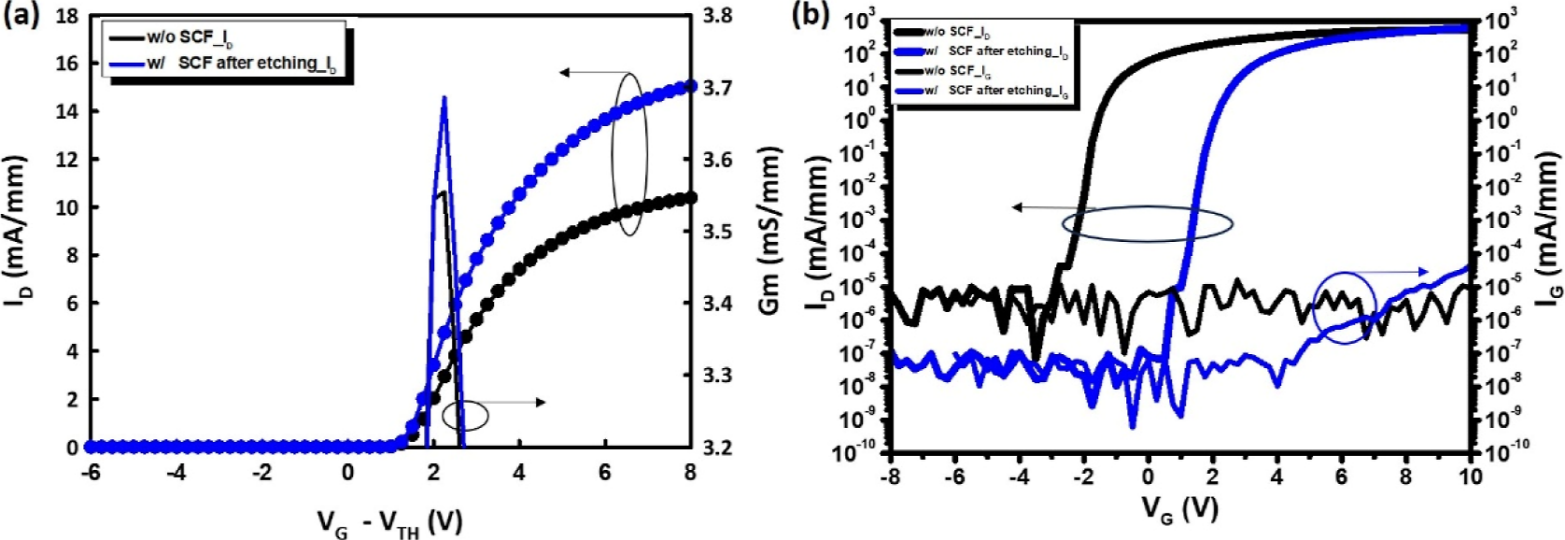
Transfer characteristics of normally off AlGaN/GaN MISHEMTs with recessed gate: (a) *I*_D_–*V*_G_ and *G*_m_ at *V*_D_ = 0.1 V. (b) Semilog *I*_D_,*I*_G_–*V*_G_ characteristics at *V*_D_ = 10 V, showing reduced gate leakage and an enhanced on/off ratio after SCFNP.

**Table 1:** Characteristics of AlGaN/GaN MISHEMT without and with SCFNP Treatment

items/SCFNP treatment	without SCFNP (*V*_D_ = 10 V)	with SCFNP (*V*_D_ = 10 V)
*I*_D,max_ (mA/mm) at *V*_G_ = 10 V	524	600
*I*_D,off_ (mA/mm) at *V*_G_ = −8 V	6.1 × 10^–6^	1.2 × 10^–7^
*I*_G_ (mA/mm) at *V*_G_ = −8 V	4.1 × 10^–6^	9.9 × 10^–8^
*V*_TH_ (V) at 1 mA/mm	–1.8	2.2
*G*_m,max_ (mS/mm)	73.3	97.6
subthreshold swing (mV/dec)	152.8	132.5
on/off ratio	8.6 × 10^7^	5.0 × 10^9^

To further evaluate the influence of SCFNP treatment on the gate dielectric interface, capacitance–voltage (*C*–*V*) measurements were performed on MIS capacitors with and without SCFNP, as shown in Figure 4(a). The SCFNP-treated sample exhibits a steeper capacitance transition in the *C*–*V* characteristics and a pronounced suppression of the secondary step, which is commonly associated with interface trap-related responses at the Al_2_O_3_/AlGaN interface. These features indicate a mitigation of interface trap-related effects and an overall improvement in dielectric/semiconductor interface quality. In addition, a positive shift in the flatband voltage (Δ*V*_FB_) is observed after SCFNP treatment, indicating a modification of the net interfacial charge condition near the AlGaN surface. This behavior reflects a redistribution and partial neutralization of interface-related charges that affect the electrostatic potential at the gate dielectric interface. Together, the steeper *C*–*V* transition, suppression of the secondary step, and the Δ*V*_FB_ shift provide quantitative electrical characteristics indicating reduced interface trap-related effects at the Al_2_O_3_/AlGaN interface after SCFNP treatment. Consistently, the MIS capacitors exhibit a positive Δ*V*_TH_ shift of approximately 1.4 V. This shift can be interpreted as an effective modulation of the net interfacial charge, estimated using Δ*Q*_net_ = *C*_ox_ × Δ*V*_TH_, corresponding to a sheet charge density on the order of 4.5 × 10^12^ cm^–2^. This net charge modulation is consistent with the suppressed Ga–O-related bonding states and enhanced Ga–N bonding observed in XPS, both of which are known to influence the electrical activity of interface states. Overall, these results indicate improved gate electrostatics and enhanced *V*_TH_ stability resulting from SCFNP-induced interfacial modification.

**Figure 4:**
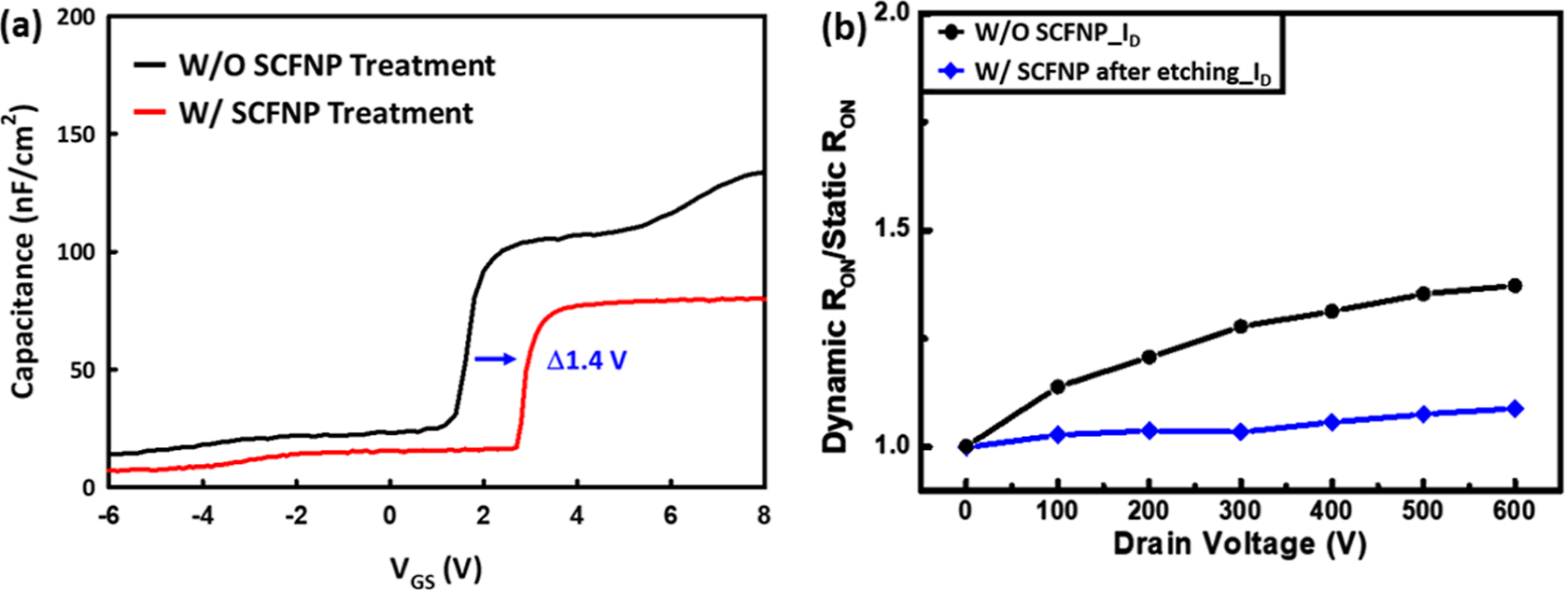
(a) Capacitance–voltage characteristics of MIS capacitors with and without SCFNP, demonstrating improved interface quality and suppressed trap-related features. (b) Dynamic *R*_ON_ characteristics under pulsed high-voltage stress, showing reduced current collapse after SCFNP treatment.

Dynamic *R*_ON_ degradation is a critical reliability concern in recessed-gate AlGaN/GaN MISHEMTs, mainly arising from electron trapping at surface and interface states during high drain-bias stress. The trapped charges partially deplete the 2DEG, leading to an increased *R*_ON_ and reduced current conduction when the device is switched back to the ON state. To investigate this phenomenon, pulsed *I*–*V* measurements were performed by holding the device in the OFF state at *V*_G_ = −10 V while applying drain bias values from 0 to 600 V for 0.1 ms. The untreated device exhibited a progressive increase in *R*_ON_ with increasing *V*_DS_, indicating pronounced charge trapping and associated current collapse. In contrast, the SCFNP-treated recessed-gate MISHEMT showed much lower *R*_ON_ dispersion across the same voltage range. At a drain voltage of 600 V, the SCFNP device exhibited a 25.6% reduction in current collapse compared to its untreated counterpart, as illustrated in Figure 4b. This observation confirms that SCFNP effectively passivates trap states responsible for high-field charge capture. By mitigating the dominant trap states contributing to charge capture under high-field switching conditions, the SCFNP process helps to maintain 2DEG stability under dynamic operation. Overall, these results demonstrate that SCFNP improves not only static leakage suppression and *V*_TH_ control but also dynamic device reliability. Therefore, SCFNP can be considered a practical postfabrication surface and interface engineering approach to address one of the key challenges in recessed-gate AlGaN/GaN MISHEMT technology.

The OFF-state BV of the recessed-gate AlGaN/GaN MISHEMT was significantly improved after SCFNP treatment, increasing from 557 to 712 V, corresponding to a 27.8% enhancement, as shown in Figure 5. All BV measurements were performed on devices with identical gate–drain spacing and field-plate geometry, and BV was defined using the same leakage current criterion to ensure a fair comparison. The observed BV enhancement is correlated with SCFNP-induced modification of the AlGaN/GaN interface, which suppresses surface- and interface-related leakage pathways under high drain bias. Cross-sectional TEM confirms the formation of an ultrathin interfacial modification layer (0.91 nm), as shown in Figure 2e. While this layer does not significantly alter the dielectric thickness, it is associated with improved interfacial conditions that are consistent with enhanced high-field insulation behavior. The presence of this interfacial modification layer is expected to promote a more uniform lateral electric-field distribution under high drain bias, thereby mitigating localized electric-field crowding near the drain-side gate edge. Such field crowding is commonly associated with surface damage and unsaturated dangling bonds introduced during recessed-gate etching, which can act as preferential sites for premature breakdown. Overall, these results demonstrate that SCFNP is an effective postfabrication passivation method that enhances high-voltage robustness and improves reliability-relevant electrical stability in recessed-gate AlGaN/GaN MISHEMTs for power applications.

**Figure 5:**
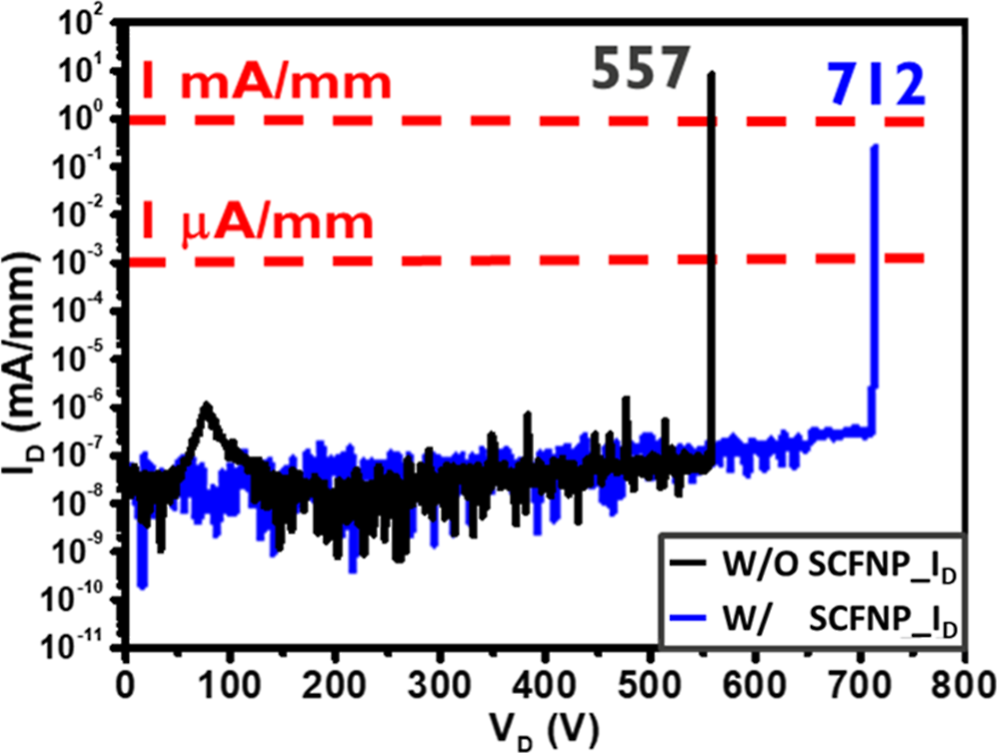
OFF-state BV characteristics of recessed-gate AlGaN/GaN MISHEMTs with and without SCFNP. SCFNP-treated devices show a significant BV improvement from 557 to 712 V at *V*_G_ = −10 V with a gate-to-drain spacing of 10 μm.

To further support the experimental findings, device simulations were performed using Silvaco TCAD,^23^ with the simulated structure consistent with the fabricated device structure configuration. The spontaneous polarization, piezoelectric polarization, and sheet carrier density were calculated using the Albrecht polarization model.^24^ In TCAD studies, breakdown behavior is typically analyzed using physics-based models that account for electric-field redistribution and high-field carrier generation mechanisms.^25,26^ In this work, the Shockley–Read–Hall (SRH) recombination model was employed to describe carrier recombination processes, while the Selberherr (SELB) impact ionization model was incorporated to account for high-field carrier generation and carrier multiplication under large reverse drain bias conditions.^27,28^ Acceptor-type traps were introduced in the GaN layer, while donor-like traps were placed at the Al_2_O_3_/AlGaN interface according to previous electrical measurements and cross-sectional TEM observations. Based on the proposed SCFNP mechanism, NH_2_^+^ species interact with surface dangling bonds, weakening Ga–O bonds and improving interface stoichiometry, which leads to a reduction in interface trap density. The baseline trap density was set to 5 × 10^17^ cm^–3^, while the SCFNP-treated case was modeled with a reduced trap density of 1 × 10^17^ cm^–3^. These values were chosen as effective parameters to reproduce the experimentally observed electrical trends and to remain consistent with the relative reduction in Ga–O bonding indicated by XPS analysis, rather than to represent absolute *D*_it_ values. Trap-related recombination was described using the Shockley–Read–Hall (SRH) formalism, and the electron capture cross section, thermal velocity, and density of states were adopted from the literature.^29^

The simulated device structure is shown in Figure 6a, and the detailed recessed-gate configuration is illustrated in Figure 6b. The OFF-state BV simulation was carried out under unified bias conditions (*V*_GS_ = −10 V and *V*_DS_ = 500 V) for both devices to compare the effect of different defect densities on the electric-field distribution. Figure 6c,d presents the simulated electric-field profiles before and after SCFNP treatment, respectively. In both cases, the maximum electric field is located at the drain-side gate edge, followed by the drain access region, which is consistent with the typical electric-field distribution in lateral AlGaN/GaN MISHEMTs under OFF-state high-voltage bias. Importantly, the order of the electric-field distribution remains unchanged before and after SCFNP treatment. However, the magnitude of the peak electric field at the drain-side gate edge is significantly reduced in the SCFNP-treated device, indicating effective mitigation of localized electric-field enhancement near the gate edge. This pronounced reduction in the peak electric field at the drain-side gate edge after SCFNP treatment directly contributes to delayed source-to-drain breakdown, consistent with the experimentally observed improvement in OFF-state BV.

**Figure 6:**
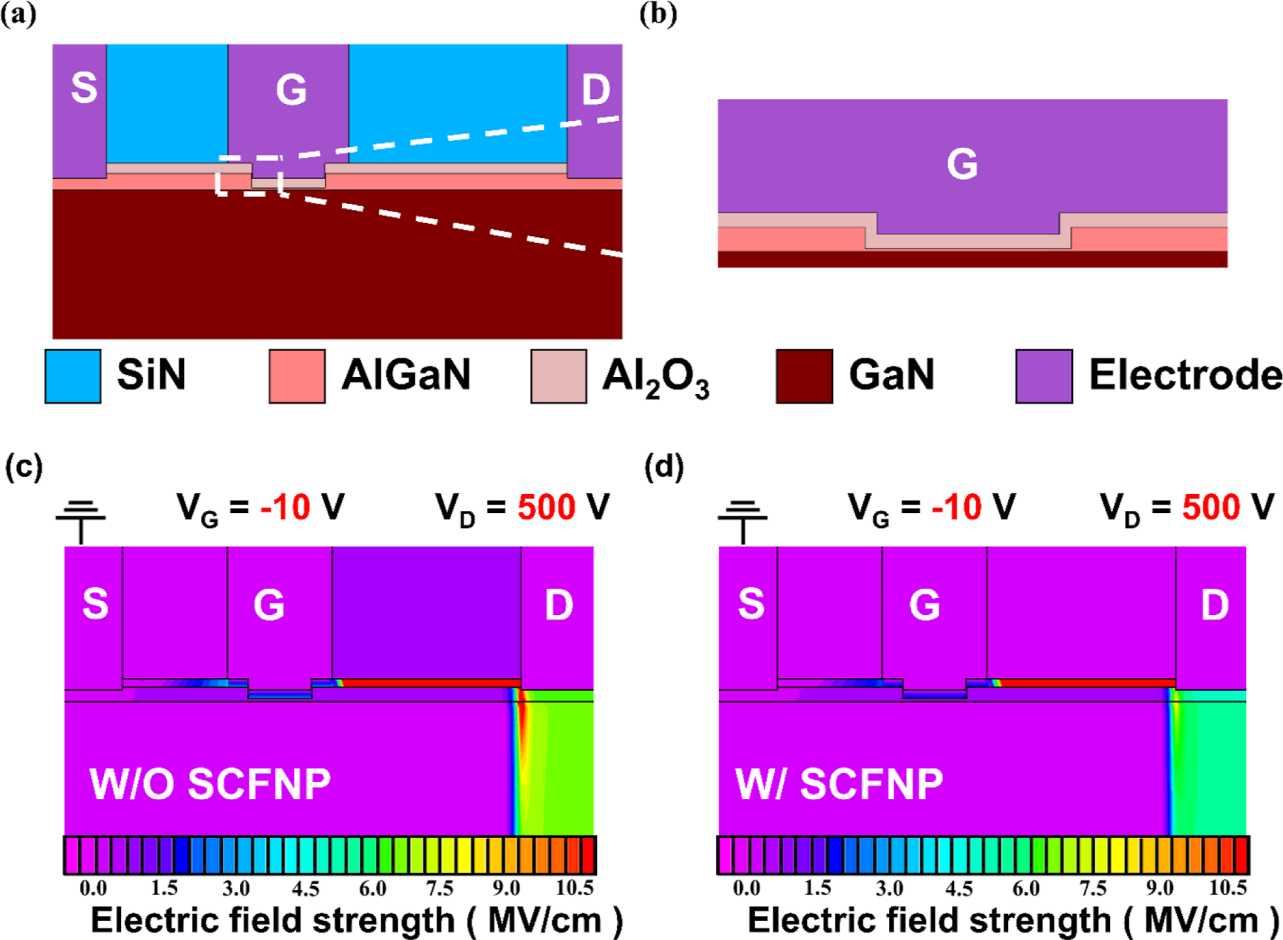
(a) Schematic diagram of the recessed gate MISHEMT. (b) Detailed illustration of recessed gate structure. Cross-sectional electric field distributions of (c) W/O SCFNP and (d) W/SCFNP.

To directly correlate the electric-field modulation with breakdown behavior, simulated OFF-state breakdown characteristics are provided in Figure 7(c),(d). The simulated OFF-state BV characteristics for the device without SCFNP yield a breakdown voltage of approximately 571 V, which is in good agreement with the experimentally measured value of 557 V, thereby confirming the validity of the breakdown modeling approach, as shown in Figure 7(c). Furthermore, Figure 7(d) compares the simulated BV characteristics with and without SCFNP treatment under identical bias conditions. The SCFNP-treated device exhibits a clear increase in simulated BV from 571 to 730 V, which is consistent with the experimentally observed BV enhancement.

**Figure 7:**
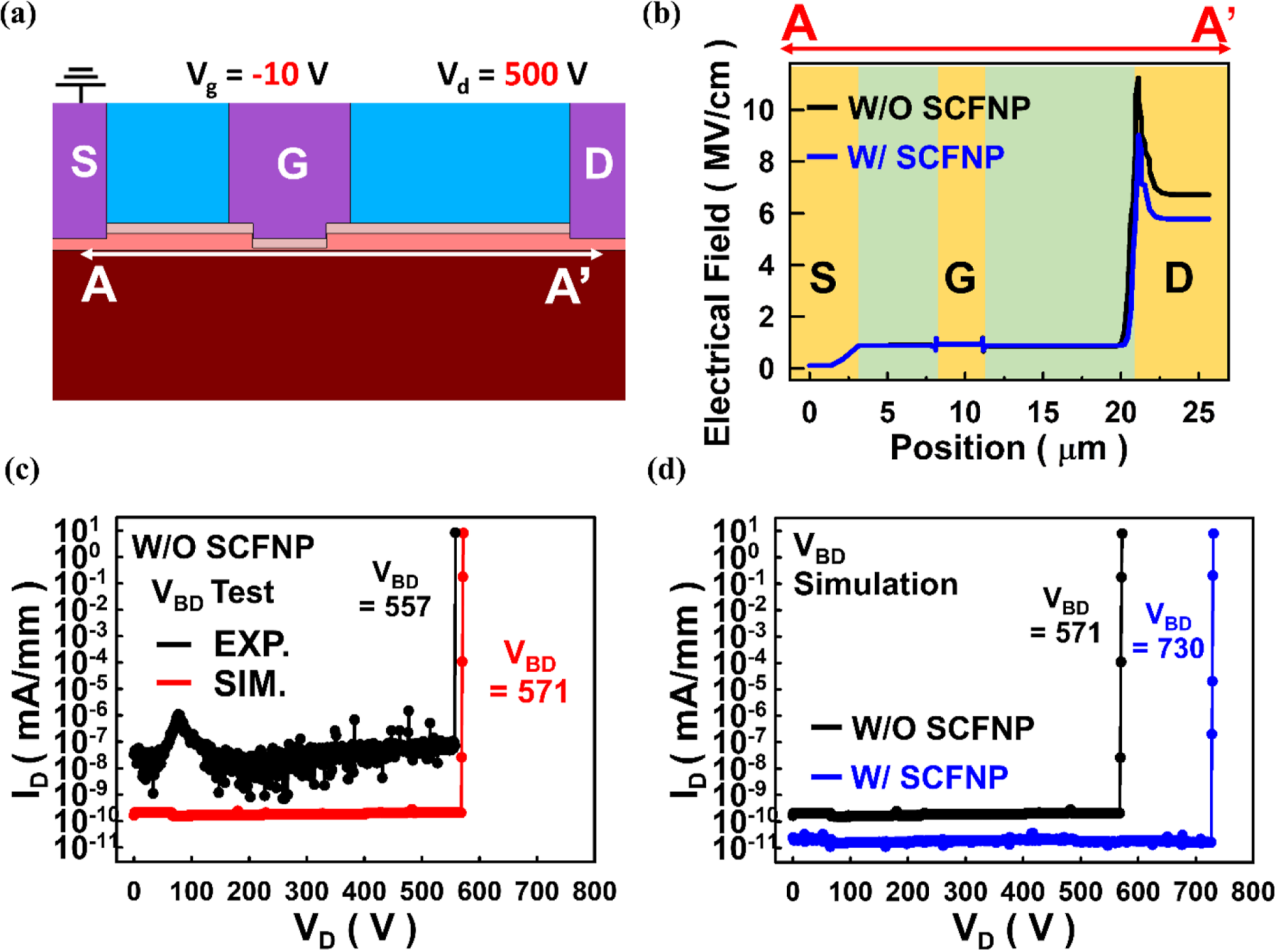
(a) S–D directional cut-line view. (b) Electric field intensity curve. (c) Comparison of experimental and simulated OFF-state BV characteristics without SCFNP treatment. (d) Comparison of with and without SCFNP treatment at simulated OFF-state BV characteristics.

These results collectively demonstrate that SCFNP effectively redistributes the electric field in the gate–drain region, thereby enhancing the high-voltage reliability of recessed-gate AlGaN/GaN MISHEMTs. The consistency between the simulated and experimental BV results further validates SCFNP as an effective approach for improving the high-voltage performance of recessed-gate AlGaN/GaN MISHEMTs.

## 4 Conclusion

This study demonstrates that SCFNP can significantly improve both the electrical performance and reliability of recessed-gate AlGaN/GaN MISHEMTs. In particular, the process provides a low-temperature (180 °C) and high-efficiency approach for repairing surface and interface defects, thereby addressing one of the fundamental challenges associated with recessed-gate structures. After SCFNP treatment, the devices exhibited a pronounced positive *V*_TH_ shift to +2.2 V (extracted at *I*_D_ = 1 mA/mm), together with an increased maximum drain current of 600 mA/mm, a substantially reduced gate leakage current of 9.9 × 10^–8^ mA/mm, and a high on/off current ratio of 5.0 × 10^9^. The gate leakage current was reduced by nearly 2 orders of magnitude, and the OFF-state BV increased by 27.8% (from 557 to 712 V). In addition, dynamic *R*_ON_ measurements indicate that SCFNP mitigates trap-related effects during OFF-state stress, resulting in a 25.6% reduction in current collapse under high-voltage operation. These improvements are correlated with the formation of an ultrathin interfacial modification layer, which is associated with improved interfacial conditions and mitigated trap-related effects under high-field operation. TCAD simulations provide qualitative insight consistent with the experimental trends, showing that SCFNP-induced interfacial modification is associated with mitigated trap-related effects and reduced electric-field crowding near the drain-side gate edge. Overall, SCFNP provides a low-temperature and scalable postfabrication passivation strategy, offering a practical solution for enhancing device efficiency and robustness against trapping-related degradation in GaN-based power devices.
